# The Inferred Cardiogenic Gene Regulatory Network in the Mammalian Heart

**DOI:** 10.1371/journal.pone.0100842

**Published:** 2014-06-27

**Authors:** Jason N. Bazil, Karl D. Stamm, Xing Li, Raghuram Thiagarajan, Timothy J. Nelson, Aoy Tomita-Mitchell, Daniel A. Beard

**Affiliations:** 1 Department of Molecular and Integrative Physiology, University of Michigan, Ann Arbor, Michigan, United States of America; 2 Biotechnology and Bioengineering Center, Medical College of Wisconsin, Milwaukee, Wisconsin, United States of America; 3 Division of Biomedical Statistics and Informatics, Department of Health Sciences Research, Mayo Clinic, Rochester, Minnesota, United States of America; 4 Departments of Medicine, Molecular Pharmacology and Experimental Therapeutics, and Mayo Clinic Center for Regenerative Medicine, Rochester, Minnesota, United States of America; Semmelweis University, Hungary

## Abstract

Cardiac development is a complex, multiscale process encompassing cell fate adoption, differentiation and morphogenesis. To elucidate pathways underlying this process, a recently developed algorithm to reverse engineer gene regulatory networks was applied to time-course microarray data obtained from the developing mouse heart. Approximately 200 genes of interest were input into the algorithm to generate putative network topologies that are capable of explaining the experimental data via model simulation. To cull specious network interactions, thousands of putative networks are merged and filtered to generate scale-free, hierarchical networks that are statistically significant and biologically relevant. The networks are validated with known gene interactions and used to predict regulatory pathways important for the developing mammalian heart. Area under the precision-recall curve and receiver operator characteristic curve are 9% and 58%, respectively. Of the top 10 ranked predicted interactions, 4 have already been validated. The algorithm is further tested using a network enriched with known interactions and another depleted of them. The inferred networks contained more interactions for the enriched network versus the depleted network. In all test cases, maximum performance of the algorithm was achieved when the purely data-driven method of network inference was combined with a data-independent, functional-based association method. Lastly, the network generated from the list of approximately 200 genes of interest was expanded using gene-profile uniqueness metrics to include approximately 900 additional known mouse genes and to form the most likely cardiogenic gene regulatory network. The resultant network supports known regulatory interactions and contains several novel cardiogenic regulatory interactions. The method outlined herein provides an informative approach to network inference and leads to clear testable hypotheses related to gene regulation.

## Introduction

Reverse engineering of a gene regulatory network (GRN) is an inverse problem that remains a significant challenge [1]–[5]. Despite high-throughput gene expression data obtained from methods such as some modified real-time PCR assays [6], high-density DNA microarrays [7], [8] and RNA Seq [9], complex interactions embedded in GRNs often overwhelm current methods of network inference [10], [11]. Thus, there exists a need for new systematic tools to aid in the identification of the underlying architecture in regulatory networks [12], [13].

A general approach to reverse engineering of GRNs involves clustering genes into hierarchical functional units based on correlations in expression profiles [14]. To infer the causal relationships between these functional units, time-lagged correlation analysis is often employed [15], [16]. Other identification methods include genetic algorithms [17], neural networks [18], Bayesian models [19], and meta-analysis approaches [20]. Several additional methods have been suggested to infer GRNs from expression data using prior knowledge of the GRN, perturbation responses, and other techniques (for details, see [4], [10], [21]–[24]). Most of these methods rely on linear relationships to reconstruct the network without considering any combinatorial effects, noise or time delays; therefore, these approaches fail to capture any nonlinear interactions and interdependencies within the network [25]. General measures of dependency based on mutual information have been used to capture these interactions in gene expression patterns [26]–[30]; however, mutual information does not give interaction directions and requires a significant amount of initial data. To circumvent these issues, a new approach that relies on a combination of linear and nonlinear relationships to account for the dynamic nature of biology was developed [31]. Though the approach was validated with *in silico* data, the present study represents the first large-scale application to a dataset derived from a biological process such as cardiogenesis.

Cardiogenesis is the process in which the mesoderm of the embryonic blastocysts forms the fetal heart through a series of transformations (for review, see [32], [33]). Morphology of heart development is well documented, but it is unclear how gene products regulate this process *in vivo*. With high-throughput technologies in genome-wide expression profiling, recent work has begun to address this complex transformation and identify key cardiopoietic factors that commit embryonic stems cells towards the cardiac-lineage genetic program [34], [35]. Gene dosage drives protein expression and normal development as evidenced by knockdown experiments. A survey of copy-number variable cardiac developmental genes has shown an enrichment of perturbed gene dosage in human children with congenital heart defects [36], emphasizing the role of molecular expression levels in dynamical networks. Going beyond curated candidate genes and identifying novel gene-gene interactions is a complimentary strategy to prioritize high value targets that may be overlooked with strategies relying on *a priori* annotations. The challenge is to determine how those key molecules come together in systems level analyses to create a fully functional organ.

We detail an approach to reverse engineer the cardiogenic gene regulatory network using a unique network inference algorithm [31]. Time-course microarray data from developing mouse hearts described in Li et al. [37] were input into the inference algorithm to obtain cardiogenic gene regulatory networks. The networks were tested against an independent, professionally curated dataset. In all test cases, maximum performance of the algorithm was achieved when the purely data-driven method of network inference was combined with data-independent, functional-based association method. The approach is performed in two phases. First, a purely data-driven network inference algorithm is used on a subset of genes to construct an informative network that is then pruned to reveal the most likely GRN that best characterizes the input data. Second, this network is used as a scaffold to include additional genes from the entire dataset. A final filtering step yields a reduced network of maximum confidence. This expanded network best characterizes the cardiogenic gene regulatory network as inferred by our algorithm.

## Materials and Methods

### Generalized Network Inference Method

**Figure 1:**
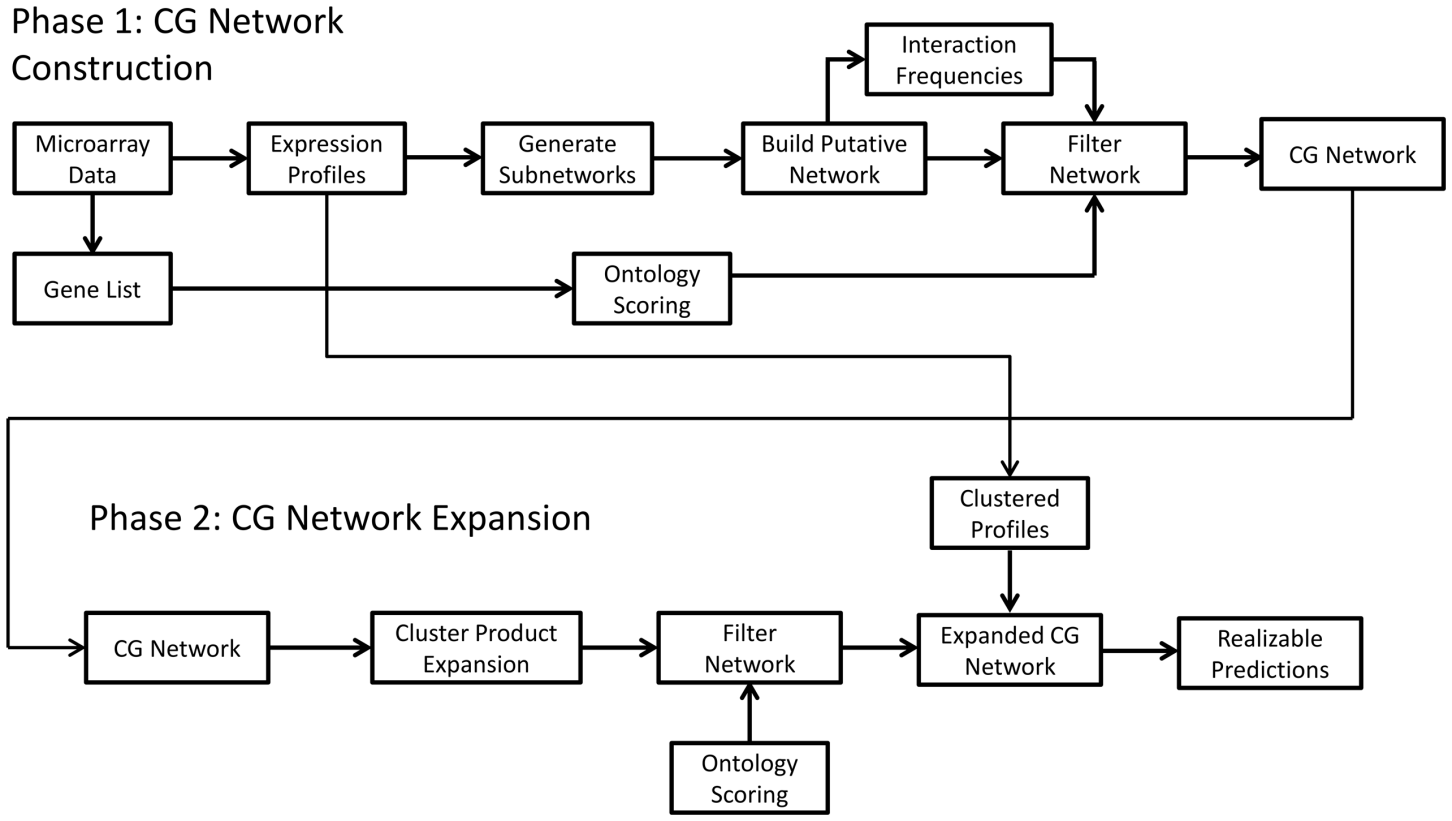
Network inference method flowchart. Phase 1 consists of constructing a scaffold network using a set of chosen genes thought to sufficiently represent the cardiogenesis process. Two independent metrics based on the interaction frequencies generated by the inference algorithm, the confidence metric, and gene ontology, the semantic similarity metric, are used to filter the network and remove spurious interactions. Phase 2 involves expansion of this scaffold network using a cluster expansion technique to produce a more complete network that best characterizes the regulatory interactions during cardiogenesis as inferred from the data. The gene interactions are further prioritized using a gene-profile uniqueness metric, the cluster product, to generate an experimentally realizable set of predictions.

A flowchart of general network inference method employed is depicted in Figure 1. The overall approach consists of two phases. Phase 1 constructs a network scaffold based on a set of genes that is assumed to capture the cardiogenesis process. The microarray data for a chosen set of genes is processed by extracting the profile data and gene lists. Independently, the expression profiles are clustered using self-organizing maps, the profiles are input into the model-based network inference algorithm [31], and the gene list is scoring using an ontology-based method [38]. When the network reconstruction algorithm returns the raw network, it is filtered using the two independent metrics called the confidence metric and the semantic similarity metric. The confidence metric is derived from the interaction frequencies determined from the model-inferred network topology. (Network topology is defined as the structure in which nodes, or genes, are connected with each other to form a network.) The semantic similarity score is obtained from the ontology scoring using GO terms that describe gene function. The two metrics form a weighted sum called the fidelity score. Phase 2 expands this scaffold network by using the clustered profiles to include other genes that were not included in the original analysis. The network is filtered again using the fidelity score. This results in an expanded cardiogenesis network that contains many more genes and interactions and is expected to capture more of the gene regulatory interactions during cardiogenesis. Predictions are prioritized using a cluster product metric, obtained from the expression profile clustering. More details about these three metrics are given below.

### Cardiogenesis Data

**Figure 2:**
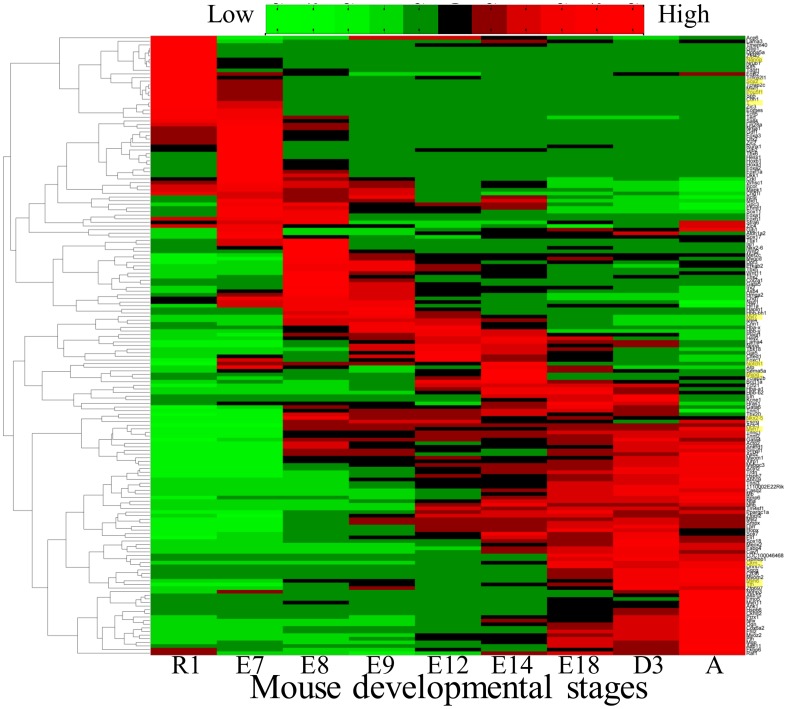
Hierarchical clustering of the mouse heart gene expression input dataset. After E8, the data are representative of gene expression in the left ventricle. The cardiogenic program is seen to propagate through the network yielding elevated expression of the typical cardiomyocyte markers by the Adult stage. The CG list profiles were clustered using the MATLAB clustering algorithm using the Pearson correlation and complete linkage metrics. The rows corresponding to the example genes for the known early stage transcription factors, *Oct4*, *Nanog*, *Sox2* and *T*, developmental genes, *Nkx2–5*, *Myl7*, *Notch1* and *Myog*, and ventricular cardiac specific markers, *Ttn*, *Myh6*, *Myh7* and *Ckm*, are highlighted in yellow on the right.

We used data obtained elsewhere that consist of gene expression at sequential heart developmental stages in mouse measured with Affymetrix Mouse Genome 430 2.0 microarrays (GSE51483). For details, see Li et al. [37]. The data are published as raw and processed formats. Gene expression data were calculated using the RMA algorithm [39] at nine developmental stages consisting of embryonic stem cells (ESCs as starting point), early and late embryonic stages until the adult stage. At embryonic day nine (E9.5) and later, the left and right ventricles were separated, and gene expression was assayed for each side. The data used in this study were only from the left side. This microarray contains 45,000 probesets representing known genes in the mouse genome. This large number prohibits the use of methods of inference that rely on model simulation with current computational capabilities and algorithms. Therefore, in order to complete the network inference, a list of genes of interest was generated from the entire mouse genome expression data based on the following selection criteria: i) the top 50 differentially expressed genes ii) the top 50 differentially expressed transcription factors and iii) a list of cardiac specific genes that are believed to be involved with a variety of congenital heart diseases [40]. The final list consisted of 171 genes (herein, the cardiogenesis list, or CG list). The expression data for these genes are used in Phase 1 network generation. The raw data are located in Table S1. Figure 2 shows a heat map of the expression for the CG list across all nine time points. For modeling purposes, probeset yielding the highest dynamic range was chosen, and the nonnegative RMA-normalized data are scaled between zero (minimum expression) and one (maximum).

### Network Inference

**Figure 3:**
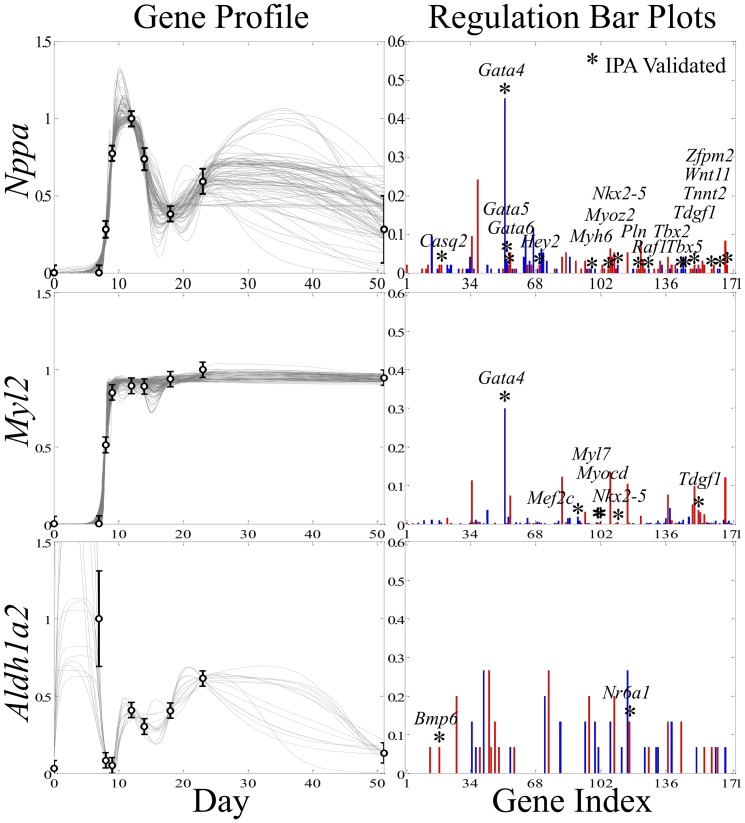
Example results from the subnetwork analysis. The algorithm returns data-consistent, simulated gene expression profiles that often show some degree of dynamical uncertainty between the data. Each line represents a separate model simulation with varied network topology. The corresponding regulation bar plots show gene-gene interaction frequencies. The height of each bar represents the fraction a given regulator appears in the subnetwork ensemble and reflects a measure of confidence for the gene interaction. Stars labeled with a gene name represent IPA validated interactions. Top panels are for *Nppa*, the middle are for *Myl2* and the bottom are for *Aldh1a2*.

Our previously described algorithm [31] was used to model the expression levels of the genes in the CG list during the development of the heart. In brief, the network inference algorithm splits an N-dimensional problem into N 1-dimensional problems, one for each observed state variable (gene). Putative regulatory networks associated with each of the individual state variables are independently identified. Network identification for each variable/gene is based on a generalized model of gene expression dynamics accounting for competition between activation and inhibition from all other genes in the dataset. Since this problem is typically under-determined, ensembles of putative subnetworks are developed for each gene. A subnetwork is a type of subgraph that only contains the target node and the neighboring regulatory nodes. Each ensemble contains anywhere from 50 to 2,000 subnetworks that support data-consistent simulations. A data-consistent simulation is defined as one that leads to a variance-weighted least squares error function less than 0.75. (See Figure 3 for examples.) Putative regulatory networks for the full 171-gene list were generated by randomly sampling and combining the subnetworks. In total, 1,000 putative networks were generated and statistical information of the gene interaction pairs, or edges, regarding frequency of occurrence, directionality, and regulatory strength (activating versus inhibiting) was collected. The full set of statistical metrics on all predicted gene pairs is given in Tables S2 and S3. (See Doc S1 for details about Tables S2 and S3.) Subnetwork generation and network analysis was done using MATLAB R2013b (The Mathworks, Inc.). Network analysis was done using the toolbox published by MIT's Strategic Engineering Research Group [41]. For network visualization, Cytoscape 3.0.2 was used [42]. Gene ontology (GO) annotations for mouse were obtained and analyzed using the GO biological process terms (download on 1/15/14) using ClueGO [43] with GO term fusion turned on and the rest of the options at the default settings.

### IPA Validation

To benchmark the network inference algorithm, a database of accepted gene regulatory interactions is required. Ingenuity Pathway Analysis (IPA) was utilized (Ingenuity Systems, www.ingenuity.com) as an expert-curated gene interaction database that is regularly updated and maintained. While the regulatory interactions in IPA are not complete, it provides a database to verify results from our inferred networks. It is important to note that a putative interaction *cannot* be ruled out because it does not appear in the IPA database as many have yet to be discovered. Exported regulatory interactions from IPA lack directionality, so analysis was done treating the network as an undirected graph. Network enrichment was calculated as hypergeometric using the hygecdf function in MATLAB. We use a hypergeometric model with N = (171*170)/2 possible edges, 396 of which were found in IPA. The interaction list for each gene in the CG list was downloaded from the IPA servers no later than 07/24/2012.

### Gene Ontology Semantic Similarity

A scoring of each predicted gene-gene interaction is computed to enable pruning our predictions by estimating the pair's biological relevance. Semantic similarity scores were calculated using pre-propagated GO terms for the mouse genome obtained from Gemma [44] using the method described in Mistry and Pavlidis [38]. Gene Ontology is a hierarchically-structured, controlled vocabulary, and most genes have multiple GO annotations. The pre-propagated annotations give a single path from the GO Biological Process root to the gene's most specific leaf node allowing the exact term set to become a proxy for the gene's biological role. A GO term set-intersection for any pair of genes quickly yields a biological similarity metric (between 0 and 100%). These are used during the filtering steps to choose those pairs believed to share a common biological process.

### Expression Profile Clustering

As the network inference uses only numerical profiles and is agnostic to each gene's true identity, common profiles will confound results. Stochastic clustering using self-organizing maps (SOM) is used to cluster gene profiles [45], [46]. As the clustering is sensitive to initial parameters, many iterations are performed and a count of gene-gene co-clustering is collected. The SOM forces all genes into a grid layout, varied randomly in size from 3×3 through 50×50 to achieve a balance of precision and smoothing. A total of 10,008 genes with stable, non-dynamic expression (defined as dispersion, or standard deviation over the mean less than 20%) are considered too common and are excluded from the SOM evaluation. This leaves 11,307 genes with sufficient dynamical expression to include in the analysis. A total of 2,240 SOMs were computed and pooled together to determine a given gene pair's coincidence frequency. Gene profiles with similar time-course dynamics will often cluster together and have high co-incidence scores. A threshold of 70% was used to partition the 21,315 gene set, resulting in 4,099 clusters. There were 2,808 singletons, so the number of clusters was further reduced to 1,291 clusters with more than one gene. The number of genes in each cluster is approximately exponentially distributed.

### Fidelity Score

A metric to gauge the fidelity of a predicted interaction is constructed to maximize biological relevance (Equation 1). The confidence metric is derived from the topological frequency distributions obtained from the network algorithm. The GO term overlap semantic similarity metric is represented by the Jaccard index [47]. Both measures are orthogonal metrics and log-normally distributed. As such, the fidelity score for *k*th gene pair, Z*_k_*(*w*), is the weighted sum of the z-scores of the log of the confidence metric, Z*c_k_* and the Jaccard index, *Zj_k_*. Only the non-zero Jaccard indices are z-scored. The z-scores for the zero Jaccard indices are set to min *Zj* - 1. This ensures that the Jaccard index z-scores are centered at zero while still giving semantically dissimilar interactions a low score. The equation to compute the fidelity score is:



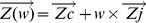
(1)where the arrow represents vector notation defined for the set of all edges. The weight for the Jaccard indices was optimized by maximizing the performance metrics. By maximizing *Z_k_*(*w*), the *k*th gene pair is more likely to reflect a true interaction and shares a high degree of semantic similarity. In other words, gene pairs that possess large fidelity scores are the most relevant predictions.

### The Cardiogenic Network

The network constructed from the initial dataset was expanded from 171 to include additional genes (Phase 2). We first constructed an eigengene network analogous to the method by Langfelder and Horvath [48]. An eigengene network consists of a network of unique gene modules that best characterize the network in a reduced, non-redundant form. A gene module is a set of genes with highly similar expression profiles. Our gene modules are derived from the clusters inferred by the SOM co-clustering frequencies. Each predicted interaction from Phase 1 is expanded to include all combinations of genes with common expression profile. For example, if gene A is predicted to interact with gene B, but gene A has four additional genes with matching profile and gene B has six, a total of five times seven gene pairs could be represented by the numerical prediction. This expanded set is ranked and filtered using Equation 1 at the optimal filter setting (*w* = 1).

## Results

### Subnetwork Ensembles Predict Regulatory Interactions

The gene profiles in the CG list were organized using hierarchical clustering. Figure 2 shows the clustered expression levels for the 171 genes for the nine time points from the beginning of development in the embryonic stem cell stage (R1) to the adult stage (A). The data show that the known pluripotent transcription factors (e.g. *Oct4*, *Nanog*, *Sox2* and *T*) peak at the early stages of heart development. During development, known cardiogenic genes activate (e.g. *Nkx2–5*, *Myl7*, *Notch1* and *Myog*). At the adult stage, ventricular cardiac specific markers (e.g. *Ttn*, *Myh6*, *Myh7* and *Ckm*) are significantly expressed. This dataset provides a natural roadmap of the dynamic gene expression patterns that synchronize cardiac maturation and is used to predict genes previously unrecognized as cardiogenic contributors.

All 171 gene profiles for the CG list were input into the network inference algorithm to produce 171 subnetwork ensembles (one for each gene) capable of explaining the expression data as interpreted by the model. (See the Network Inference subsection in the Methods for details.) Figure 3 highlights a few typical examples of the model simulations and topological frequency distributions produced by the network inference algorithm. These results demonstrate some common features of the subnetwork ensembles. First, all model trajectories pass through or near the experimental data. Without additional data, each model simulation presented is equally valid. Second, in some instances, the model simulations do not significantly vary (e.g. *Myl2*, where each simulated expression profile is overlapping). Third, the predicted dynamics can vary considerably between time points as shown by the *Nppa* and *Aldh1a2* examples. This is dynamical uncertainty is a result of the model parameter estimation and introduces a unique opportunity for the design of optimal experiments (for details, see [49], [50]). Typically each subnetwork ensemble contains hundreds simulations that are data-consistent. However, some subnetwork ensembles contain less than 100 that are considered acceptable (e.g. *Aldh1a2*). In this case, the algorithm had trouble finding combinations of regulatory interactions capable of fitting the data. There are only approximately 50 different model simulations presented for this example. While each subnetwork ensemble varies in the population size and dynamical uncertainty, they all support data-consistent simulations. Thus, they all represent the possible regulatory interactions for the true cardiogenesis GRN.

The regulation bar plots in Figure 3 (right) reflect the topological frequency distributions for the *Nppa*, *Myl2* and *Aldh1a2* subnetwork ensembles. Genes are on the x-axis and labeled with their index. The height of each bar represents how often the network inference algorithm found that particular interaction sufficient to support a data-consistent simulation. For example, *Gata4* appeared as an activator 45 times per 100 putative subnetworks for *Nppa*, and the height of the bar for *Gata4* is 0.45. In other words, the height reflects how confident the algorithm is at calling a particular interaction as real given the input expression profiles. These heights are defined as the confidence metric and are used for filtering. For the *Nppa* and *Aldh1a2* examples, the dynamical uncertainty shown in the simulated expression profiles is associated with the many low frequency potential regulators seen in their corresponding regulation bar plots and arises from many unique topologies that could explain the data.

The labeled stars signify regulatory interactions found in the IPA database. For 11% of the genes, the highest ranking regulator identified by the algorithm is reported in the IPA database. Among the population of all regulators identified by the algorithm, 6% are reported. In some cases, the highest ranked regulators returned by the algorithm are not previously reported and are thus targets for experimental validation. For *Nppa* and *Myl2*, the network inference algorithm identified a likely regulator, *Gata4*, and other possible regulatory interactions. Many of these additional interactions may be interpreted as being noise; however, among this noise are 14 (for *Nppa*) and 5 (for *Myl2*) regulatory interactions found in IPA. The case of *Aldh1a2* shows a slightly different scenario. The number of interaction detected is lower than for *Nppa* and *Myl2*, and the algorithm did not detect any dominant regulatory interactions.

### Algorithm Performance Measures

**Figure 4:**
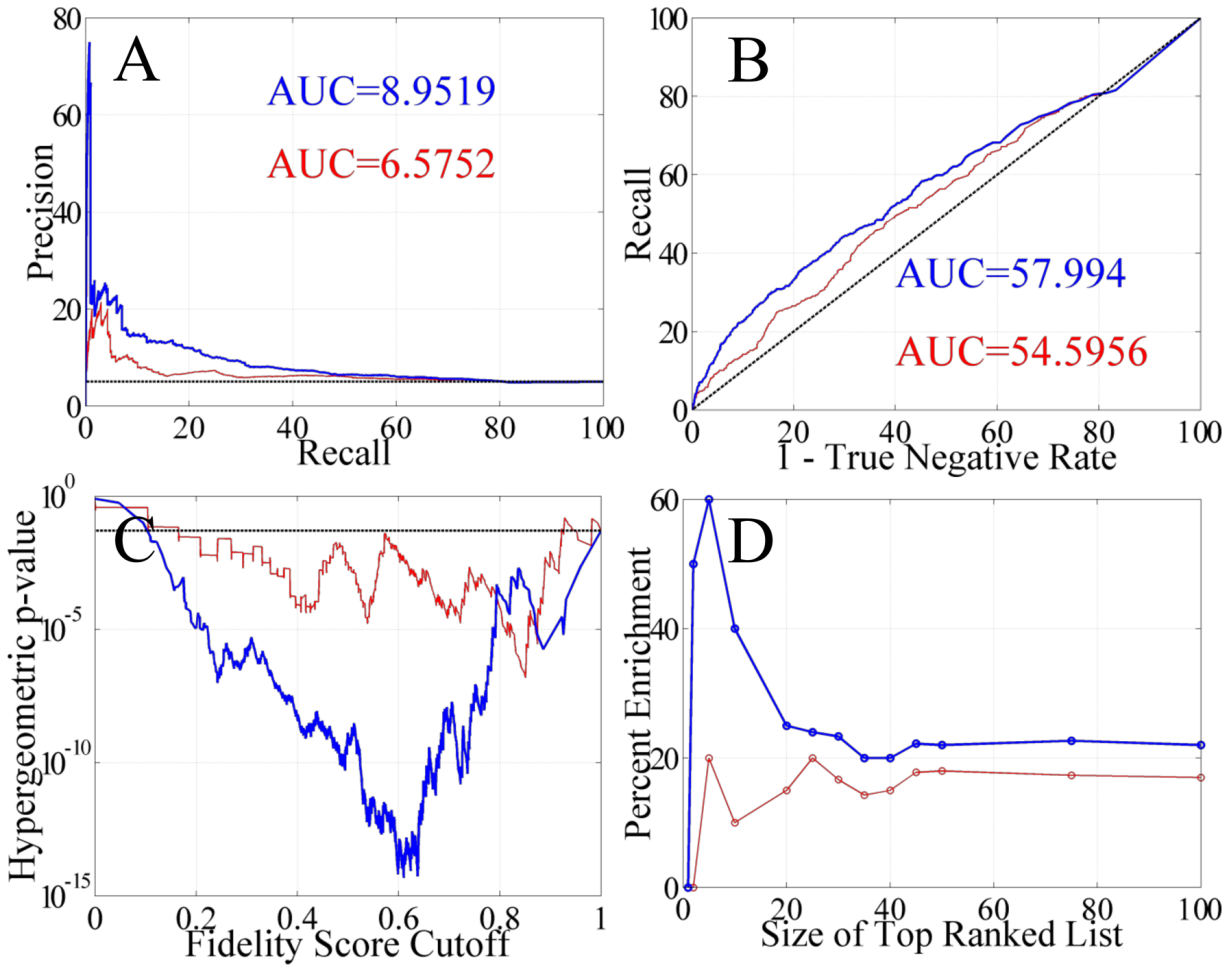
Performance metrics of the algorithm using a network created using the IPA database. The precision-recall curve (A), receiver operating characteristic curve (B), the significance level of filtered networks (C), and the degree of IPA enrichment in the top ranked set of genes (D) are shown for two different types of filters. The fidelity scores were linearly scaled to facilitate comparison between the two filters. The red line represents the results obtained when filtering the networks with only the confidence metric defined by ***Z***(0). The blue line shows the results obtained when the networks are filtered with both the confidence score and the semantic similarity score using ***Z***(1). In all cases, the networks filtered with both metrics (blue) produced superior networks relative to those filtered with only the confidence score (red) or the semantic similarity score (not shown). The dotted lines represent random prediction for (A) and (B) and the 0.05 significance level for (C).

The likelihood that a given regulatory interaction is represented in the true cardiogenesis GRN is assumed to be proportional to i) the frequency that that interaction appears in the subnetwork ensembles and ii) the degree of overlap or similarity in the GO term annotations. These two metrics combine to form a weighted sum called the fidelity score (Equation 1). When the weight, *w*, is zero, the score consists of only the confidence metric. For *w* >> 1, the score is dominated by the semantic similarity metric. For 0>*w*>10, the score reflects a mixture of these two metrics. Removing edges with a low fidelity score makes it possible to explore the algorithm's performance measures using the IPA network as a comparison. Based on optimizing the performance of the algorithm, *w* = 1. This indicates that both the confidence metric and the semantic similarity metric are of equal importance. For these tests, only genes that were sufficiently annotated in IPA were included in the analysis to avoid any offset bias. The performance measures examined are shown in Figure 4. It must be noted that due to the incomplete nature of IPA, this approach only determines a lower bound of performance.

The typical performance measures reported with network inference algorithms are the precision recall (PR) curve and the receiver operating characteristic (ROC) curve [51]. These measures reflect the overall performance of the algorithms with the PR curve being the most unbiased. The average performance for each measure is given by the area under the curve (AUC). It is evident that the fidelity score that combines the confidence and semantic similarity metrics produces the best results. In Figure 4A, the PR curve is shown with an AUC score of 9% with both metrics but 6.6% when only the confidence metric is used. As the predicted network interactions are pruned via the filtering process, a higher fraction of true interactions are retained giving higher precision values at lower recall levels. In Figure 4B, the ROC curve is shown with an AUC score of 58% with both metrics but 55% when just the confidence metric defines the fidelity score. This indicates that the algorithm does an acceptable job when comparing the recall (sensitivity) versus the rate of identifying a negative as a false negative (1 – specificity). Overall, these types of behavior are expected as the algorithm is purposefully biased towards minimizing false negatives at the expense of false positives. The justification behind this design is that it is easier for the experimentalist to remove a false positive via experimentation using the predicted topology versus searching in an unknown topological space for false negatives. Although, it is not possible to directly compare the algorithms' performance metrics with other published algorithms, it is possible to make a rough comparison with the inference algorithms that participated in the DREAM5 challenge [11]. The AUPR and AUROC scores are better than those published in the DREAM5 challenge for the *S. cerevisae* network. (See the YEASTTRACT row in Figure S2 and the *S. cerevisiae* column in Figure S3, in Supplementary Notes 3 and 4, respectively, of Marbach et al. for comparison [11]). Note that the dataset used in the challenge contained much more data than available in this study's mouse heart gestational time-course.

Additional performance measures such as the network significance and true positive enrichment of the highest ranked interactions are presented in Figure 4C and 4D, respectively. The fidelity scores are linearly scaled to facilitate comparison between the two filters, ***Z***(0) and ***Z***(1). Recall that ***Z***(0) are the fidelity scores computing with only the confidence metrics and ***Z***(1) are the fidelity scores computing using both the confidence metrics and the semantic similarity metrics with a weight, *w* = 1. For either filter, significant networks are recovered as the fidelity cutoff is increased as shown in Figure 4C. With no fidelity filtering, the initial network has 12,084 edges (323 found in IPA). Although 81.6% are found, this is not significant due to ‘calling’ 83% of all possible edges. At extremely high fidelity cutoff values, fewer interactions are retained and correspondingly few edges found in IPA. With the semantic similarity metric included in the fidelity score, many false positives are removed from the network. However, 146 out of 396 true positives have a zero semantic similarity. This hinders the filtering process at low fidelity scores, but the concomitant removal of false positives at higher fidelity scores compensates and results in a superior filter. Filtering the networks with both metrics also increases the percent of true positives in the top ranked edge lists as shown in Figure 4D. With either filter, the percentage of true positives in the list of edges with the highest fidelity scores asymptotically approaches 20%. The combined filter yields much higher enrichment for the smaller ranked lists up to the top 20 ranked edges.

### Further Testing of the Algorithm

The algorithm was also tested using two networks constructed from the IPA database. The semantic similarity metric was used to choose 50 genes that were expected to interact with one another and 50 genes that were not. Herein, these two networks will be referred to as the IPA enriched and IPA depleted networks. The IPA enriched network consists of 283 validated interactions while the IPA depleted network consists of only 3. In general, the algorithm returned denser and more significant networks for the IPA enriched network versus the IPA depleted network. When comparing the two inferred networks, the confidence metric is used since it is an absolute measure. Fidelity scores are derived from z-scores and cannot be used to compare networks.

**Figure 5:**
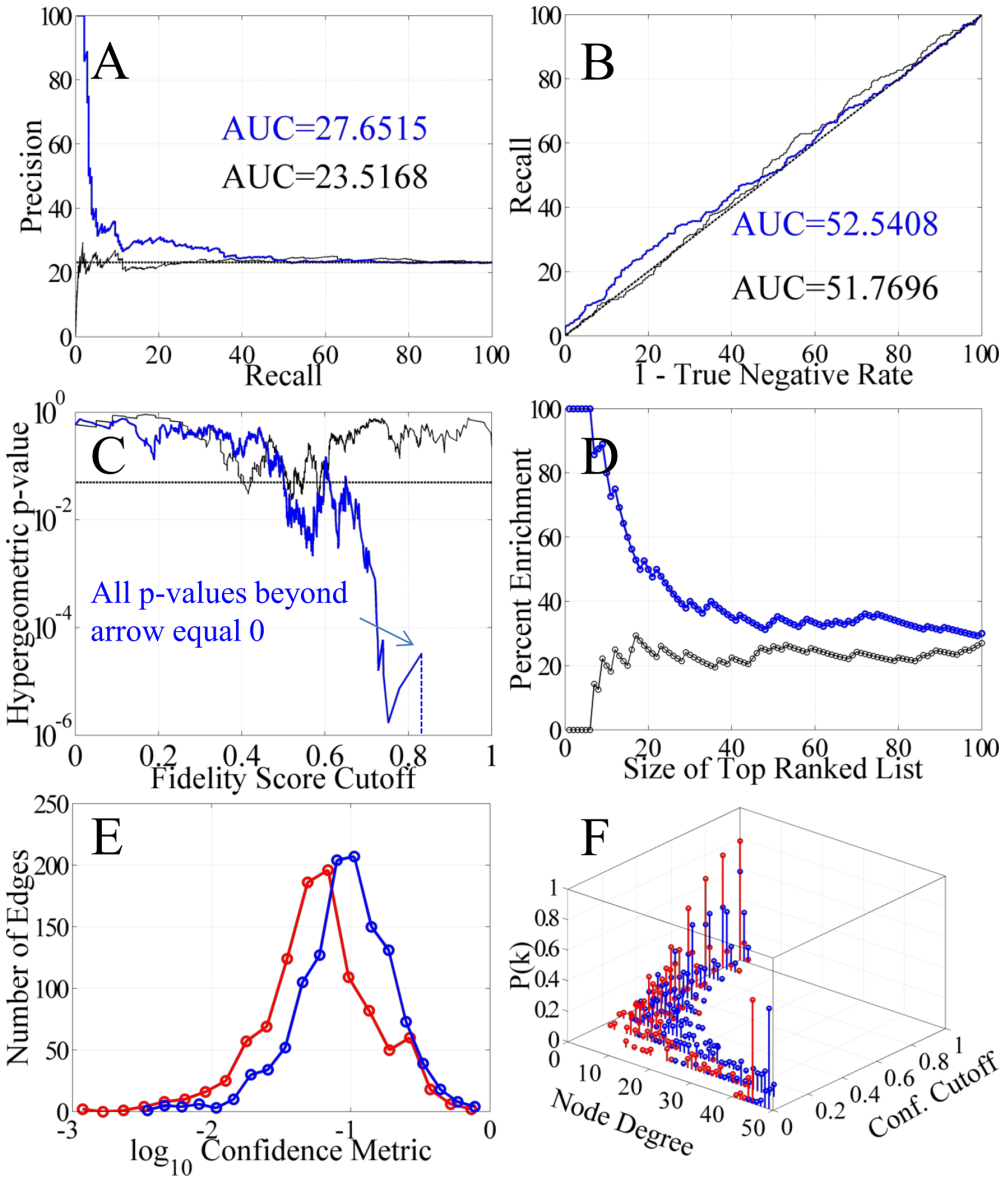
Performance on networks known to be enriched with true interactions *a priori* and depleted of true interactions. The precision-recall curve (A), receiver operating characteristic curve (B), the significance level of filtered networks (C), the degree of IPA enrichment in the top ranked set of genes (D), number of edges inferred (E), and the connectivity distributions at varying confidence score thresholds are shown for two different types of filters for the IPA enriched and IPA depleted networks. The fidelity score cutoff was normalized to facilitate comparison between the two filters. The black line represents the results obtained when filtering the networks with only the confidence metric for the IPA enriched networks. The blue line shows the results obtained when the networks are filtered with both the confidence metric and the semantic similarity metric using the optimal filter settings for the IPA enriched networks. The red line, when present, signifies the results obtained when using the networks filtered with the optimal filter settings. All genes in the IPA depleted network had semantic similarity metrics of 0, so this metric cannot be used to filter the network. For (C), the missing segments after the vertical dotted line correspond to p-values = 0. For the (A) and (B), the results obtained for the IPA depleted networks are not shown due to the sparse IPA validated interactions. The dotted lines represent random prediction for (A) and (B) and the 0.05 significance level for (C).

Performance measures for the networks inferred from the IPA depleted network are not shown as only three edges are true in the reference network. The AUPR and AUROC for the inferred IPA depleted networks are 3.2% and 55%, respectively. The corresponding values assigned to random chance are 2.9% and 50%, respectively. Therefore, the algorithm returns better results compared to random chance. Figure 5A and 5B show that when the optimal filter settings were used, the AUPR and the AUROC scores for the networks inferred from the IPA enriched network were also superior to random chance. All possible IPA validated interactions are all present in the unfiltered networks. Applying the filter produces statistically significant networks as shown in Figure 5C. But only at the optimal filter settings and high fidelity score cutoffs are statistically significant networks returned by the algorithm. Also, when the fidelity score cutoff was high, the algorithm also returned statistically significant networks for the IPA depleted network. The percent IPA interactions in the top ranked list are significantly increased when the optimal filter settings are used as shown in Figure 5D. By exploiting the semantic similarity metric, many false positives are pruned from the network and IPA validated edges are enriched in the highest ranked predicted interactions.

Initially, both inferred networks contained nearly the maximum amount of total possible edges. This is by design so as to minimize false negatives. (See Algorithm Performance Measures section for details.) They begin to take on dramatically different topologies as they are filtered. As shown in Figure 5E, the IPA enriched inferred network contains more edges with higher confidence metrics. As such, the network retains more edges relative to the IPA depleted inferred network as the networks are filtered. This leads to denser, reconstructed networks and is confirmed by observing the connectivity distributions as shown in Figure 5F. For a given confidence metric cutoff, the node degree distribution for the IPA enriched inferred networks is almost always to the right than the networks inferred from the IPA depleted networks. This demonstrates that the networks inferred from the IPA enriched networks were highly interconnected.

### Filtering Network Using Confidence Metrics Reveals Scale-free, Hierarchical Networks

**Figure 6:**
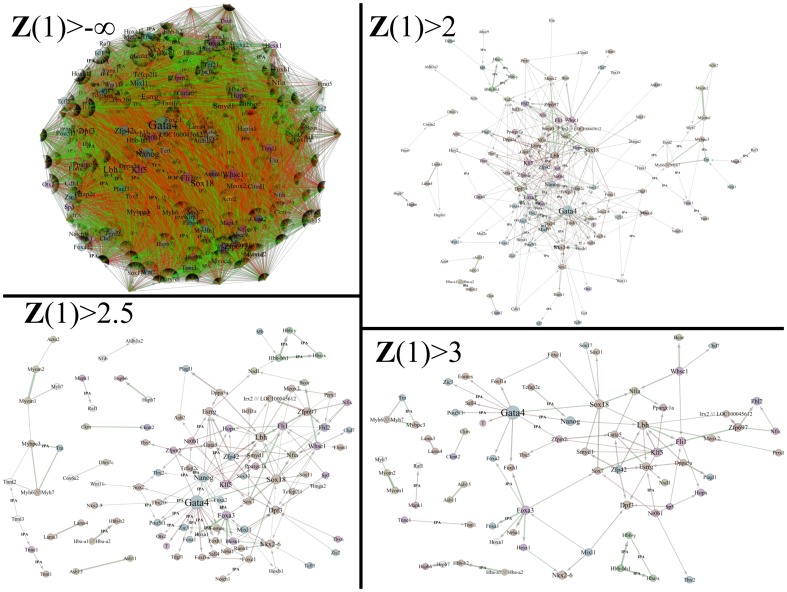
Predicted gene regulatory network of 171 nodes at filter cutoff values of -∞, 2, 2.5, and 3. The complete network shows the ‘hairball’ characteristic of an exponential network. Interactions also found by *Ingenuity Pathway Analysis* are marked with ‘IPA.’ Edge thickness represents the confidence score. Edge color is red for inhibiting, green for activating, and yellow for unclear relationships. Node edges are colored according to their ontological ID. See Figure 7 for the GO term legend. Node sizes and labels are scaled with the node degree. As the cutoff metric is raised, scale-free, hierarchical networks emerge.

**Figure 7:**
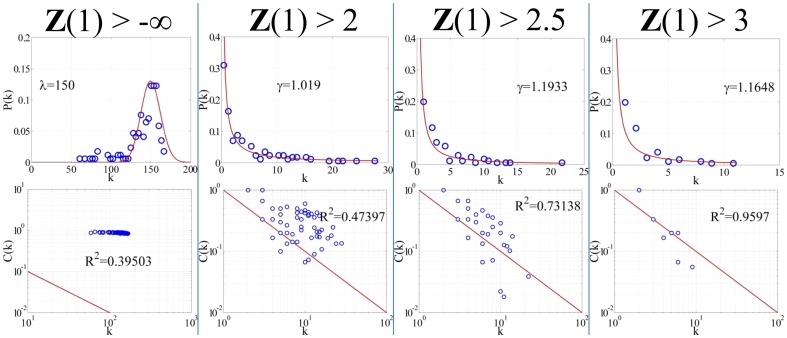
Node degree and clustering coefficient distributions. When the filtering cutoff is set to -∞, all edges returned by the inference network are retained and form a hairball which is characteristic of an exponential network. The connectivity distribution (top) follows a Poisson distribution with λ equal to 150, while the clustering coefficient distribution (bottom) is flat and independent of the node degree. As the cutoff metric is applied to the network, scale-free, hierarchical networks emerge. At a cutoff of 2, the connectivity distribution follows a power law with γ equal to 1.02, and the clustering coefficients begin to scale with the reciprocal of the node degree with an R^2^ value of 0.47. At an even more stringent cutoff value of 2.5, the network further represents a scale-free, hierarchical network where γ equal to 1.19 and R^2^ equal to 0.73. At a cutoff value of 3, the network becomes even more hierarchical with an R^2^ value of 0.96.

Constructing a network from all the regulatory interactions identified by the network inference algorithm generates a highly connected ‘hairball’ network that closely mimics an exponential network [52]. However, by removing edges with low fidelity scores, the topology drastically changes. This is demonstrated in Figures 6 and 7. Figure 6 shows several example networks generated using fidelity score thresholds; while, Figure 7 shows the corresponding topological measures. Initially, the network contains all 171 genes linked together with 12,084 edges (83% of all possible connections). The node degree distribution follows a Poisson distribution with λ ∼ 150. The clustering coefficient distribution is flat and independent of the node degree, *k*. More informative networks are deduced from this hairball by pruning away the low fidelity scoring edges. As the score threshold is increased, a more familiar topology is revealed. With a moderate threshold enforced (***Z***>2), the node degree distribution follows a power law distribution with γ equal to 1.02. The clustering coefficient distribution is a function of the inverse of the node degree with an *R* ^2^ value of 0.47. This network consists of 118 genes linked together with 346 edges. With a stricter threshold placed on the fidelity score (***Z***>2.5), the node degree distribution still follows a power law distribution, but γ increases to 1.19. Similarly, the clustering coefficient distribution remains a function of the inverse of the node degree, except that the *R* ^2^ improves to 0.73. The resulting network has 99 genes connected together with 169 edges. At an even stricter threshold (***Z***>3), the network is scale-free with γ equal to 1.16, and it becomes even more hierarchical in structure with an *R* ^2^ value of 0.96. In this network, there are only 31 genes linked together with 89 edges. Thus, as the confidence score cutoff is increased, the inferred networks possess a scale-free topology and hierarchical structure [52]. If the threshold is set too high, the network becomes disjoint and topological measures are inapplicable. These networks are available as a Cytoscape file, Network S1 (see Supporting Information).

### Highest Scoring Interacting Genes are Enriched with Known Interactions

Of the high scoring networks presented in Figure 6, the single most important gene based on regulatory interactions is *Gata4*. This gene is involved in a wide variety of processes involving embryogenesis, cardiogenesis, and muscle development [53]. It is critical for the proliferation and maintenance of cardiac tissue [54], [55]. And many of the genes downstream of Gata4 were successfully predicted by the algorithm. Moreover, a few genes further downstream that are recovered from the algorithm and shown in the ***Z***>2.5 filtered network are also in the IPA database. These include *Nkx2–5*, *Wnt11*, and *Fhl2*. *Nkx2–5* is a gene involved with cardiac hypertrophy and embryonic stem cell pluripotency [56]. It is linked to *Gata4* via *Tbx20*, a gene associated with the maintenance of functional and structural phenotypes for the heart [57]. *Wnt11* is connected to *Gata4* via *Sox2*. Both of these genes are important for embryonic development [58]. *Fhl2* is involved with cell adhesion, mobility and survival [59] and is connected to *Gata4* via *Nanog*, *Lbh* and *Fli1*. *Nanog* is a known pluripotent transcription factor [60] and may be involved with the preservation of pre-committed lineages for proliferation during organogenesis facilitated by Lbh [61] and *Fli1* [62], [63]. The examples presented corroborate the interactions identified by the algorithm and suggest that the predictions made by the algorithm are worth experimentally pursuing. Four of the top ten gene interactions identified here have already been validated as shown in Table 1. *Fli1* is of particular interest because when the secondary filter (cluster product filter) is applied, it is enriched in the top ranked predictions in Table S2, as well as, in the expanded network as shown in Table S3.

**Table 1:** Top 10 Gene Interactions from the CG List.

Gene-Gene Interaction	Fidelity Score	Validation
Myom1 interacts with Myom2 via activation	5.65	-
Hbb-bh1 strongly regulates Hbb-y via activation	5.19	IPA
Hba-x is strongly regulated by Hbb-bh1 via activation	4.84	IPA
Hba-a1///Hba-a2 strongly regulates Hbb-b2 via activation	4.80	IPA
Foxa3 strongly regulates Nr6a1 via activation	4.77	-
Foxa1 is strongly regulated by Foxa3 via activation	4.34	IPA
Lama3 regulates Lama4 via inhibition	4.18	-
Fli1 is strongly regulated by Sox7 via activation	4.16	-
Sox18 interacts with Sox7 via activation	4.15	-
Foxa3 strongly regulates Foxh1 via activation	4.11	-

Fidelity scores were computed using Equation 1 with *w* = 1.

### Network Expansion Reveals Novel Regulatory Modes

Although the input data (CG list) consist of less than 200 cardiac related genes, Figure 2 shows that the expression of early transcription factors in the embryonic stem cell stage and heart tube activates a wave of gene expression that leads to the sustained expression of adult cardiomyocyte related genes. Assuming this dataset captures this phenomenon reasonably well, it is possible to reach into the entire mouse dataset [37] and identify a representative cardiogenic GRN in the mammalian heart. The initial network returned by the algorithm was used as a scaffold and the entire mouse heart dataset (consisting of more than 20,000 genes) was utilized to expand the network. The expanded network consists of 1,080 genes and 63,558 edges as given in Table S3. The corresponding networks are included as a Cytoscape file, Network S2 (see the Supporting Information).

**Figure 8:**
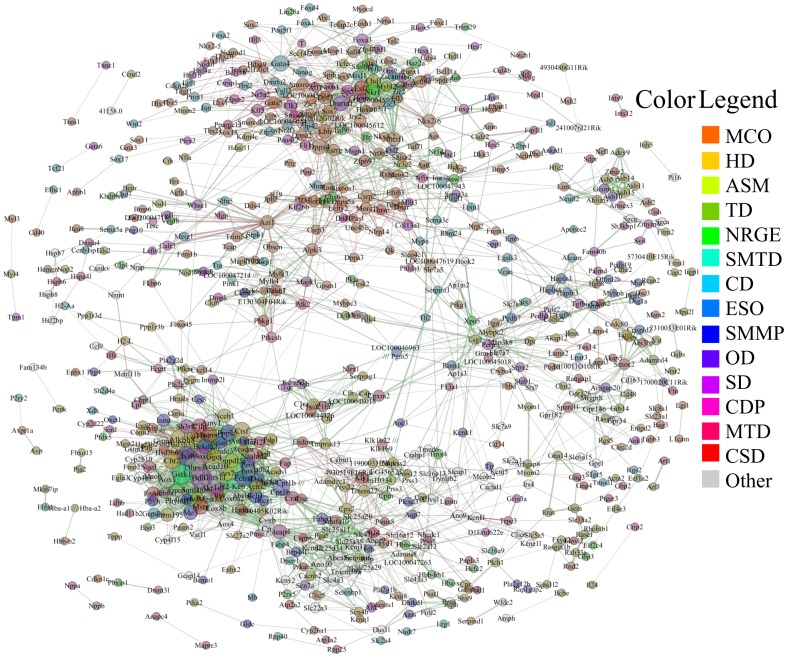
Network Expansion. The inferred network using the CG list was used as a scaffold and extended to include genes from the entire mouse genome by expression profile similarity. Representative annotations using the Gene Ontology database are shown by node color. All annotations are relevant to cardiogenesis with some more specific than others. Edge color and thickness are as in Figure 3. Directional arrows are omitted for clarity. The gene interactions shown are the edges with fidelity scores greater than 2.5. GO term acronyms: MCO, multicellular organismal development; HD, heart development; ASM, anatomical structure morphogenesis; TD, tissue development; NRGE, negative regulation of gene expression; SMTD, striated muscle tissue development; CD, cell differentiation; ESO, extracellular structure organization; SMMP, small molecule metabolic process; OD, organ development; SD, system development; CDP, cellular developmental process; MTD, muscle tissue development; CSD, cardiovascular system development; O, other. White nodes have no annotation ascribed. See also Tables S2 and S3.

The highest ranked edges of this network are shown in Figure 8. This network is filtered down to 740 genes and 2,942 edges by removing edges with Z<2.5. It best characterizes the cardiogenic gene regulatory network as inferred by the network inference algorithm. Gene annotations reveal a majority of genes are involved with embryogensis, the development of the cardiovascular system, heart morphogenesis, muscle energetics and epigenetics. The p-values for all the go terms selected using ClueGO in Table S4. Approximately 90% of the genes had representative GO annotations.

Many of the representative GO terms for this network are developmental pertaining to organogenesis, the cardiovascular system, and morphogenesis. There is a heavy cluster of genes with these annotations that consist of many transcription factors (e.g. the Fox, Gata, Tbx, Sox and Zic families) among sparsely interwoven genes involved in cell signaling, cell migration and metabolism located in the upper part of Figure 8. This cluster serves as the central network hub that connects the rest of the network and coordinates gene expression for a variety of biological processes. A small cluster of cell adhesion related genes (e.g. the Hapln family along with *Ntm* and *Pcdh7*) near the middle-right of the network. This cluster appears to be interacting with the Laminin family (proteins involved with cell adhesion, differentiation, migration and signaling) and additional proteins involved with angiogenesis, cell-cell recognition, and extracellular signaling via Srpx2, Pcdh7 and Spp1, respectively. These form an interconnected network that lead back to Lbh in the central hub via the extracellular matrix proteins *Dpt* and *Col13a1*.

In another example, a large cluster of genes heavily involved in metabolic processes is found in the bottom-left part of Figure 8. These genes mainly code for metabolic related proteins involved with CoA-mediated metabolic processes (e.g. *Acad10* and *Acadm*), mitochondrial energetics (e.g. *Cox8b* and *Cox6a2*), redox-mediated signaling (e.g. Dhrs family), oxidases (e.g. *Aox1*, *P4htm*, and *Maob*) and other metabolic processes (e.g. *Dlat*, *Ldhd*, and so on). This cluster is connected to the central hub by *Alkbh8*, a gene important for angiogenesis [64], via *Azi1*, a cell cycle related gene.

Table S3 consists of many well-characterized interactions that are important for cardiogenesis. Among them is a particularly important interaction involving *Nkx2–5* activation by *Gata4*. Durocher et al. demonstrate *Gata4* binds to the C-terminus autorepressive domain of *Nkx2–5* and activates this transcription factor [65]. Another interaction identified in the table is between *Sox18* and *Sox7*. These two transcription factors have been described to act concomitantly during cardiac and vascular development [66], [67] which suggests the existence of a mutual feedback type of regulation. Also, *Fog2* (*Zfpm2*), a cofactor of *Gata4*, is recognized as an inhibitor of *Gata4* activity [68], [69], although not necessarily of *Gata4* expression. The functional result of this interaction was also identified by the predictive network as *Zfpm2*-mediated inhibition of *Gata4*. Finally, some directionally undefined interactions such as that between *Tbx20* and *Gata4* [70], [71] are resolved in the networks, indicating a *Tbx20* activation by *Gata4*.

Among the list of genes in Tables S2 and S3, *Fli1* is the most promising candidate gene predicted to be involved in cardiogenesis targeted for experimental validation. *Fli1* is over-represented in many high confidence edges, shares a high degree of semantic similarity and shares a profile with relatively few other genes. This gene encodes a transcription factor containing an ETS DNA-binding domain and may be involved with a variety of biological processes such as cellular differentiation, proliferation, migration, apoptosis and angiogenesis [62], [63]. Although there is no direct evidence of its role in cardiogenesis that we are aware of, it is essential for embryogenesis and endothelial gene expression [72]. Furthermore, *Fli1* increased expression has been linked to decreased cardiac fibrosis in a physiological model system of cardiac damage and may imply a regulatory role not previously recognized [73]. Another interesting prediction in Tables S2 and S3 is *Tbx18* inhibiting *Sox7*; both of these genes are important early transcription factors. *Tbx18* has been shown to convert cardiomyocytes into pacemaker cells, and plays a role in tissue engineering [74]. This could be used to model cardiac pathologies such as atrial arrhythmias or ventricular arrhythmias [75] and provides *in silico* prioritization of gene therapies.

## Discussion

The approach presented herein relies on a purely data-driven inference algorithm coupled to an informative association and filtering method. In doing so, the most likely predictable gene interactions obtained from the algorithm are those appear often in the subnetwork ensembles and those that share many GO terms. Of the top 10 gene pairs identified using the CG list, four are previously known. This is a significant achievement considering that the approach is data-driven, relies on a computational model to approximate gene expression, and supplemented with an ontology library. Including the GO terms in the selection process, dramatically improved the information retrieval tests. But the optimal filter settings were when there were equal contributions from the confidence metric and the semantic similarity metric to the fidelity score.

The network generated from the CG list was expanded using gene profile similarity metrics to include approximately 900 additional genes in the mouse genome. The expanded network consists of 1,080 genes and 63,558 edges. After filtering the network using the fidelity scores, a scale-free, hierarchical network forms that represents the cardiogenic gene regulatory network as predicted by the algorithm. The network predictions are too numerous to check with rigor, and the examples shown herein that corroborate the network are just a few of the many possible plausible interactions present in the network. That said, many of the predictions are either already known or are worth experimentally validating.

The substantial boost in performance by including both the confidence metric and semantic similarity metric in the fidelity score is consistent with what others have found. Nazri and Lio found that combining their meta-analysis approach with Relevance Network [26], significantly enhanced predictive capabilities [20]. And Marbach et al. concluded that a community-based inference strategy was superior to any single method [11]. The approach presented herein applies a similar strategy by combining a purely data-driven method with a functional-based association method. The end result is superior performance. Combination of additional, independent methods would only increase performance even further.

In addition to testing the algorithm on the CG list, it was tested on two additional networks of vastly different qualities. One was enriched with known connections from IPA while the other was depleted of them. The algorithm inferred denser networks for the IPA enriched network as compared to the IPA depleted network. And as with the networks inferred from the CG list, the best results were obtained for the IPA enriched inferred networks when the fidelity score included both the confidence metric and the semantic similarity metric. From these analyses, it is clear that model-based inference part of the algorithm adequately constructs putative regulatory interactions capable of explaining the data. It may seem surprising that time course microarray data, for as much as it reveals, is information-poor. The system is too under-determined, and there are too many different, plausible ways to put the network together while still corroborating the expression data. Thus, it is important to supplement the predictions of any model-based algorithm with independent information (e.g. semantic similarity).

Methods used to construct networks using gene expression profiles are typically undermined by the similarity of the expression between various genes in the dataset. This makes assigning network edges challenging since a given regulatory interaction can be also explained by swapping out the source gene with another gene that has a very similar expression profile. Genes of this nature have been called module genes [48]. A mitigating strategy is to focus on interactions that can be explained by relatively few genes and share common pathways. This type of approach has recently been utilized to construct gene networks and shown to produce superior results when compared to more traditional methods [76]. For the approach described herein, a secondary filter was applied to the networks to remove gene interactions pairs that can be explained by a large list of possible combinations using the cluster product scores. (See Table S3 for details.) Doing so results in the discovery of *Fli1* as a cardiogenic transcription factor. By prioritizing experimental inquiries, more time and resources can be applied to testing other predictions.

Although the algorithm performs well, improvements in data quantity and quality, as well as, ontological depth and coverage are expected to significantly improve the predictive power of the algorithm. While the dataset used to generate the regulatory network is of great quality, the tissue excised from the growing hearts consists of multiple cell types which likely hinders precise network inference. The tissue is quite heterogeneous, and gene expression in the heart is region specific [77], [78]. Applying the algorithm to a dataset obtained from a more homogenous prep, such as cardiomyocytes derived from induced pluripotent stem cells, is expected to produce more relevant networks. Another problem with this approach is the fact that over half (66%) of the interactions predicted by the algorithm for the CG list had no semantic similarity score, despite 31% of them being reported in IPA. Using a more complete set of GO terms is expected to increase the performance of the algorithm even more. Finally, better profile clustering will lead to better expansion and help avoid making erroneous predictions. This requires more robust clustering algorithms and precise measurements of gene expression.

While the algorithm is among the most efficient in its class [31], it is still computationally expensive to exhaustively search all possible combinations of gene interactions. Improving the profile clustering and using more complete semantic annotations is expected to enhance the algorithm's predictive capabilities. To reduce the complexity of the inferred networks, the algorithm can be augmented to exploit additional information obtained from pathway analyses and independent data. This will lead to an algorithm that produces more experimentally testable hypotheses, result in more efficient network inference and deliver more relevant biological networks. The approach presented herein is well suited to increase our collective understanding of the processes involved with cell lineage commitment, characterize the progression of polygenic diseases, and help unravel the complexities associated with pharmacogenomics. To further validate the inference approach, the highest ranking regulatory interactions will be tested using induced pluripotent stem cells driven towards cardiomyogensis.

## Supporting Information

Table S1 Raw expression of 171 genes used in Phase 1.
(XLSX)

Table S2 Edge properties of Phase 1 cardiogenesis network.
(XLSX)

Table S3 Edge properties of Phase 2 cardiogenesis network.
(XLSX)

Table S4 ClueGO node property table for the Phase 2 cardiogenesis network.
(XLSX)

Doc S1 Description of Tables S2 and S3.
(DOCX)

Network S1 Phase 1 cardiogenesis networks.
(CYS)

Network S2 Phase 2 cardiogenesis networks.
(CYS)
