# Supplementary material for: The Inferred Cardiogenic Gene Regulatory Network in the Mammalian Heart
Source: PLoS One. 2014 Jun 27;9(6):e100842. doi: 10.1371/journal.pone.0100842 (PMC4074065; doi:10.1371/journal.pone.0100842)
Supplement: Doc S1 — Description of Tables S2 and S3. (DOCX) [file pone.0100842.s005.docx]

Description of Tables S3 and S3

Tables S2 and S3 have very similar layouts. The only difference is that Table S3 does not contain a column for IPA given interactions. They consist of a list of predicted interactions and their edge properties derived from the analysis described in the main text. Table S2 contains the predicted interactions for the set of genes identified in the CG list as described in the main text (Phase 1). Table S3 contains the list of predictions for the expanded network described in the main text (Phase 2).

Column A identifies one of the genes for a given predicted interaction. Column B describes the directionality of the interaction based on value in Column H. Column C identifies the second gene for a given predicted interaction. Column D gives the functional relationship between the two genes based on Column I. Column E gives the GeneID for the gene in Column A. Column F gives the geneID for the gene in Column C. Column G describes the edge weight associated with the data-driven component of the algorithm. These edge weights are derived from subnetwork ensembles returned by the algorithm and range from 0.001 to 1. They are related to the regulation bar plots discussed in the main text. These are log-normally distributed. Column H contains another metric derived from the subnetwork ensembles and ranges from -1 to 1. A value of -1 means that the prediction interaction is such that the gene in Column C is the source and the gene in Column A is the target. A value of 1 means that the prediction interaction is such that the gene in Column A is the source and the gene in Column C is the target. A value of 0 implies that no definitive direction could be inferred by the algorithm. Column I gives the type of regulatory interaction (activation or inhibition) derived from the subnetwork ensembles and ranges from -1 to 1. A value of -1 means that the algorithm inferred an inhibitory relationship. A value of 1 means that the algorithm inferred an interaction that is characterized by activation. A value of 0 means that the type of interaction is ambiguous. Column J defines if a given interaction is reported in the IPA data base. A value of 1 means that it is found in IPA. A value of 0 means that it is not. A value of -1 means that the predicted interaction contains at least one gene that was not in the IPA reference network. This was used to remove such interactions and avoid biasing the performance measures. Column K describes the term overlap score described in the main text. It ranges from 0 to 1 where 1 indicates a strong functional relationship and 0 means no functional relationship is known or expected. These are log-normally distributed. Column L represents the cluster product computed from the product of the number of genes in the cluster associated with the gene in Column A and the gene in Column C. These are log-normally distributed. Columns M, N and O are the z-scores of the log10 transform of Columns G, K and L, respectively. Column P is the fidelity score with TOscore weights defined in cell R(1,1) and cluster product weights defined in T(1,1). The cluster product weights are only used for experimental testing prioritization for reasons given in the main text. They were not used during the performance tests, but are integrated in fidelity score to facilitate secondary filtering using the tables.
